# Long-Term Effects of Intermittent Adolescent Alcohol Exposure in Male and Female Rats

**DOI:** 10.3389/fnbeh.2017.00233

**Published:** 2017-11-28

**Authors:** Eva M. Marco, Sara Peñasco, María-Donina Hernández, Anabel Gil, Erika Borcel, Marta Moya, Elena Giné, José Antonio López-Moreno, Consuelo Guerri, Meritxell López-Gallardo, Fernando Rodríguez de Fonseca

**Affiliations:** ^1^Departamento de Fisiología (Fisiología Animal II), Facultad de Ciencias Biológicas, Universidad Complutense, Madrid, Spain; ^2^Departamento de Fisiología Humana, Facultad de Medicina, Universidad Complutense, Madrid, Spain; ^3^Centro de Investigación Príncipe Felipe, Valencia, Spain; ^4^Departamento de Biología Celular, Facultad de Medicina, Universidad Complutense, Madrid, Spain; ^5^Departamento de Psicobiología, Facultad de Psicología, Universidad Complutense, Madrid, Spain; ^6^Unidad Gestión Clínica de Salud Mental, Instituto de Investigación Biomédica de Málaga (IBIMA), Hospital Regional Universitario de Málaga-Universidad de Málaga, Málaga, Spain

**Keywords:** alcohol, adolescence, drinking-in-the-dark, sex differences, cognitive function, neural plasticity, hippocampal formation, frontal cortex

## Abstract

Alcohol is a serious public health concern that has a differential impact on individuals depending upon age and sex. Patterns of alcohol consumption have recently changed: heavy episodic drinking—known as binge-drinking—has become most popular among the youth. Herein, we aimed to investigate the consequences of intermittent adolescent alcohol consumption in male and female animals. Thus, *Wistar* rats were given free access to ethanol (20% in drinking water) or tap water for 2-h sessions during 3 days, and for an additional 4-h session on the 4th day; every week during adolescence, from postnatal day (pnd) 28–52. During this period, animals consumed a moderate amount of alcohol despite blood ethanol concentration (BEC) did not achieve binge-drinking levels. No withdrawal signs were observed: no changes were observed regarding anxiety-like responses in the elevated plus-maze or plasma corticosterone levels (pnd 53–54). In the novel object recognition (NOR) test (pnd 63), a significant deficit in recognition memory was observed in both male and female rats. Western Blot analyses resulted in an increase in the expression of synaptophysin in the frontal cortex (FC) of male and female animals, together with a decrease in the expression of the CB2R in the same brain region. In addition, adolescent alcohol induced, exclusively among females, a decrease in several markers of dopaminergic and serotonergic neurotransmission, in which epigenetic mechanisms, i.e., histone acetylation, might be involved. Taken together, further research is still needed to specifically correlate sex-specific brain and behavioral consequences of adolescent alcohol exposure.

## Introduction

Alcohol is a serious public health concern and age and sex have been reported as main factors affecting alcohol consumption and alcohol-related harm (WHO, 2014). In particular, the use of alcohol frequently initiates during adolescence; and the adolescent brain is still undergoing maturation and reorganization programs, and responds to alcohol differently than adults. Drinking rates are often reported to be higher at adolescence as compared to adulthood (consult reviews from: Crews et al., 2016; Spear, 2016b), and alcohol use at adolescence may also increase the risk for mental illness and substance abuse disorders in adulthood (Crews et al., 2016). It is worth analyzing sex differences since an increased vulnerability of females to alcohol-related harm has been reported (Wilsnack et al., 2013).

Notably, in the last decades, alcohol consumption patterns have substantially changed. Nowadays, heavy episodic drinking—also known as binge-drinking—has become popular among the young population (WHO, 2014). Consequently, new preclinical approaches have emerged to more closely mimic the currently adopted pattern of adolescent alcohol consumption. Among those, drinking in the dark (DID), has emerged as a valuable tool in both mice (Crabbe et al., 2011) and rats (Holgate et al., 2017). DID was selected because this approach considers: (1) ethanol self-administration, closer to voluntary alcohol intake in humans; (2) intermittent access to ethanol, providing a cycle of consumption-withdrawal that has been previously related to escalating patterns of consumption; and (3) DID has been adapted to the adolescent period (see Carnicella et al., 2014; Crews et al., 2016; Spear, 2016a for review). Despite not devoid of limitations, DID has arisen as the most suitable schedule in rodents to investigate the consequences of adolescent alcohol administration.

Therefore, in the present study we have investigated the consequences of adolescent alcohol exposure, by using the DID method, in which animals are given access to ethanol (or tap water) for 2-h sessions during 3 days, and for an additional 4-h session on the 4th day.

Alcohol withdrawal signs include heightened anxiety together with a dysregulation of the hypothalamic-pituitary-adrenal axis activity and the release of glucocorticoids (Rasmussen et al., 2000, 2001; Mons and Beracochea, 2016; Somkuwar et al., 2017). Thus, short after alcohol cessation, anxiety-like responses were evaluated in the elevated plus maze (EPM) and plasma corticosterone levels measured. Adolescent alcohol has extensively been related with impairments in cognitive function later in life (Guerri and Pascual, 2010; Alfonso-Loeches and Guerri, 2011) as measured in several paradigms including the novel object recognition (NOR) test (Sanchez-Marin et al., 2017). Therefore, we evaluated animals’ recognition memory in the NOR and focused on two brain regions largely involved in recognition memory, frontal cortex (FC) and the hippocampal formation (HF) (Squire et al., 2007; Morici et al., 2015).

Although the underlying mechanisms of adolescent alcohol exposure are not completely understood, several molecular targets have been identified. In the first place, FC and HF have been frequently described as particularly vulnerable to alcohol effect (Alfonso-Loeches and Guerri, 2011; Ozsoy et al., 2013; Oliveira et al., 2015; Staples and Mandyam, 2016). In addition, changes in glial cells (Evrard et al., 2006; Kane et al., 2014; Oliveira et al., 2015) and activation of the immune response (Montesinos et al., 2016) have been reported after adolescent alcohol exposure. Alterations in the neurogenesis and/or several players of brain plasticity have also been described (Briones and Woods, 2013), as well as modifications in several neurotransmitter systems such as the serotonergic and dopaminergic systems (Crews et al., 2016). The endocannabioid system has also been given a role in the consequences of adolescent alcohol (Sanchez-Marin et al., 2017). Therefore, we scanned for several neurobiological markers related to astrogliosis (anti-glial fibrillary acidic protein, GFAP), neural plasticity (brain derived neurotrophic factor, BDNF; and pre-synaptic proteins such as synaptophysin, SYN and SNAP25), and neurotransmitter signaling systems such as dopaminergic, serotonergic, cannabinoid and other neurotransmitter systems in these two brain areas.

Epigenetic modifications occur during the developing brain and are associated with plasticity and behavior (Fagiolini et al., 2009; Roth, 2013). Indeed, epigenetic changes have been reported to underlie some effects of alcohol on brain and behavior (see Ponomarev, 2013 for review) and our previous results have demonstrated an association between behavioral changes and changes in histone acetylation (H3 and H4), in preFC of adolescent mice with binge ethanol treatment in adolescence (Pascual et al., 2012; Montesinos et al., 2016). Therefore, in the present study, acetylation changes of H3 and H4 were also investigated.

## Materials and Methods

### Animals

The animals employed were the offspring of adult *Wistar* rats purchased from Harlan Laboratories^®^ (Milan, Italy). Following 15 days of habituation animals were mated (one male with two females) for ten consecutive days, then, pregnant females were isolated and daily observed for delivery control. At birth (postnatal day, pnd 0), litters were culled and sex balanced—no cross-fostering allowed—up to eight pups per dam (4 males and 4 females); then, litters were left undisturbed until weaning (pnd 22) when rats were housed in pairs of siblings of the same sex.

Animals were housed in plastic cages (50 × 25 × 17.5 cm) at the animal facilities in the Faculty of Biological Sciences at the Complutense University of Madrid (EX08-UCS). Animals were maintained at constant conditions (temperature, 21 ± 1°C and humidity, 60 ± 10%), under a 12 h light-dark inversed cycle (light son at 20.00). Food (2018 Global Diet; Harlan Laboratories^®^) and water were provided *ad libitum* except during exposure to alcohol.

This study was carried out in accordance with European Directive 2010/63/EU and in compliance with the Spanish Royal Decree 53/2013 on the protection of animals used for research and other scientific purposes. The protocol was approved by the “*Comité de Experimentación Animal (CEA)*” of the *Universidad Complutense de Madrid* (Madrid, Spain).

### Alcohol Exposure during Adolescence

**Figure 1:**
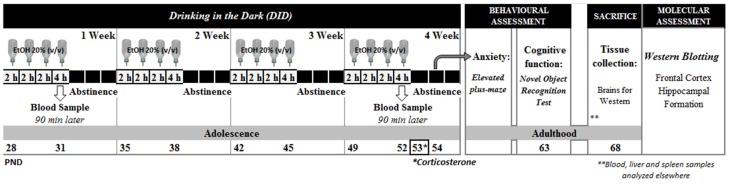
Experimental design.

As shown in Figure 1, animals were exposed to alcohol for the whole adolescence period, from pnd 28 to pnd 52 (Spear, 2000). A modified drinking in the dark administration (DID) protocol based on Crabbe et al. (2011) was employed; each week, animals were exposed for 2 h to a single bottle of an ethanol solution (20%, v/v) for three consecutive days, and for a 4 h session on the 4th day, for the following 3 days animals had no access to alcohol. The ethanol solution was prepared from ethanol 96° (Alcoholes Aroca S.L., Madrid, Spain) in tap water. For the drinking sessions animals were moved to similar plastic cages, singly housed and placed in an adjacent room. Control animals were submitted to the same manipulation although in their cages the single bottle contained tap water. Water and ethanol solutions were daily replenished.

Alcohol (or water) consumption was daily calculated by weighting bottles before and after exposure to the drinking. Body weight and food intake in the home-cage (data not shown) were also controlled throughout the administration protocol. Additional bottles with the ethanol solution and tap water were included to control for spillage and evaporation during the test sessions.

### Experimental Design

We have employed a “within-litter design”, in which all the experimental groups are represented within the same litter (Festing, 2006). A total of 12 litters were submitted to the present protocol; and within each litter, the two animals housed together were assigned to the same drug condition (control *vs*. alcohol). In the present study we will present data from the 12 litters regarding alcohol intake; four litters remained intact and were devoted to a different study. The other eight litters were used for the behavioral analysis, blood samples were collected, and at sacrifice, half of the brains were collected and employed in this study for western blot analysis, while the remaining animals were dedicated to another study which results have been already published (Pavón et al., 2016). Therefore, these neurobiological data come from animals previously submitted to behavioral analysis.

Alcohol withdrawal signs were investigated short after alcohol cessation: plasma corticosterone levels were analyzed 24 h after the last alcohol exposure session (pnd 53), and anxiety-like responses were evaluated in the elevated plus-maze (EPM) 48 h after the last alcohol session (pnd 54). Cognitive function was evaluated in the long-term, and recognition memory was evaluated in the novel object recognition (NOR) test in young adult animals (pnd 63). Then (pnd 68), animals were sacrificed by rapid decapitation. Brains were rapidly extracted and dissected on ice. Frontal cortex (FC) and Hippocampal Formation (HF) were stored at −30°C until further analysis (see Figure 1).

### Behavioral Assessment

#### Elevated Plus-Maze

The elevated plus-maze (EPM) is formed by two open arms (50 cm × 10 cm) and two equally sized enclosed arms with 40 cm high walls, arranged so that the arms of the same type are opposite one another. The junction of the four arms formed a central square area (10 cm × 10 cm). The apparatus was made of opaque black polyvinyl chloride (PVC) elevated to a height of 62 cm. On the test day, animals were placed in the central platform of the apparatus facing one of the enclosed arms, and they were allowed to freely explore the maze for 5 min under red light conditions. Whenever an animal entered an arm with all four limbs, it was considered a visit, and the frequency and duration of the visits to the open and closed arm were recorded. Some animals fell from the plus-maze and were excluded from the analysis. The percentage of open arm entries and the percentage of time spent in the open arms, considered the most relevant parameters related to anxiety, were calculated upon the total entries into any arm and upon the total time spent in both arms. Instead, the frequency of closed arm entries was considered as an index of general motor activity (Pellow et al., 1985). Between animals, the apparatus was carefully cleaned with water to remove possible odors.

#### Novel Object Recognition Test (NOR)

The NOR was performed in a square arena (60 cm × 60 cm × 45 cm) with matte-painted metallic walls and a plastic-covered wooden floor divided by white painted lines into 36 squares (10 cm × 10 cm). Animals were exposed to a 3 days habituation period to the arena, followed by the training and test session on the 4th day (pnd 63) as previously described (Ennaceur and Delacour, 1988; Mateos et al., 2011) with some minor modifications.

During the habituation period, animals were allowed to freely explore the arena, under dim light conditions, for 5 min. On the first day, the behavior of the animal in the arena was video recorded for subsequent behavioral evaluation. On the test day, *training session*, rats were first exposed to two identical objects (two plastic boxes) for them to explore the objects for at least 30 s or for a maximum period of 4 min. After a 4 h inter-trial interval, *test session*, rats were exposed to one of the previously encountered objects (familiar object, F1 or F2) and to a novel, unfamiliar object (metallic colored box, N) for 3 min. The objects were not bigger than twice the size of a rat and they were located in contiguous corners, at a distance of 10 cm from the walls. At the beginning of each session, the animals were placed in the center of the apparatus facing the wall opposite to the objects. For each animal, the position of the objects was not changed between the training and the test session. However, the objects’ position was changed between animals in order to avoid spatial preference. The apparatus and the objects were carefully cleaned between tests on different animals with a 20% (v/v) ethanol solution. Both training and test sessions were video recorded (Sony DCR-DVD310E) and the animals’ behavior was later evaluated by an experienced observer by means of event-recorder software (Observer^®^, Noldus, Netherlands). Exploration of an object was considered whenever animals pointed their nose toward an object at a distance ≤1 cm, while turning around, climbing and/or biting the objects was not considered as exploration. The time animals’ spent exploring the objects during the two sessions was registered, and the discrimination index (DI) was calculated as the difference between the time spent exploring the novel object (N) and the familiar one (F1 or F2) in relation to the total time spent exploring the objects ((N − F)/(N + F)). Animals that explored for less than 20 s during the training session and those exclusively exploring only one of the objects during the test session were excluded from the statistical analyses.

### Blood Ethanol Concentration (BEC)

Blood samples were collected from the tail vein 90 min after the 4 h session of alcohol exposure on the first and last week of alcohol exposure, pnd 31 and 52, respectively (McClain et al., 2011). Blood samples were collected into capillary tubes that contained EDTA dipotassium salt (Microvette CB 300 K2E, Sarstedt, Germany); blood was then centrifuged at 1500 rpm for 15 min at 4°C, and the plasma was stored at −20°C. BEC was determined using the EnzyChrom ethanol assay kit following the protocol recommended by the manufacturer (Bioassay Systems, Hayward, CA, USA). All measurements were performed in duplicate.

### Corticosterone Measurements

Blood samples were collected, between 10.00 and 13.00, into capillary tubes containing EDTA dipotassium salt (Microvette CB 300 K2E, Sarstedt, Germany); blood was then centrifuged at 1500 rpm for 15 min at 4°C, and the plasma was stored at −20°C. Corticosterone was measured using a solid phase ^125^I radioimmunoassay (Immuchem™ Corticosterone kit, MP Biomedicals, Orangeburg, NY, USA). The detection limit was 7.7 ng/ml and the intra-assay and inter-assay coefficients of variation were less than 10%. All samples were run in duplicate and plasma corticosterone levels were calculated from the standard curve.

### Western Blot Analysis

Only one FC and HF from each animal was randomly assigned to evaluate protein expression levels of GFAP, CB1R and CB2R cannabinoid receptors and synaptic plasticity markers in the Faculty of Medicine of the Universidad Complutense de Madrid. Neurotransmitter and epigenetic markers were measured in the Research Center “Príncipe Felipe” in Valencia.

#### GFAP, Cannabinoid Receptors and Synaptic Plasticity Markers

The tissue samples were homogenized at a ratio of 1:3 (w/v) in ice-cold lysis-buffer (Hepes 10 mM pH = 7.5; EGTA 10 mM; EDTA 10 mM; NaCl 150 mM; CHAPs 2.5%) with protease inhibitors (Roche) and PMSF 0.1 M. After homogenization, samples were centrifuged at 14,000 rpm for 20 min at 4°C. Supernatants were transferred to a new tube and the protein concentration was estimated by Bradford protein assay (Bio-Rad, Hercules, CA, USA), measure by Multiskan FC (Thermo Fisher Technologies) and analyzed by software Skanlt (Multiskan FC, version 2.5).

In each assay the same amount of protein was loaded in all wells (20 or 30 μg) depending on the protein to be detected and resolved by using 7.5%–12% SDS-acrylamide gels. After electrophoresis proteins were transferred to nitrocellulose membranes (GE Healthcare Life Sciences, UK) and transfer efficiency was determined by Ponceau red dyeing. Membranes were then blocked with Tris-buffered saline (TBS: NaCl; Tris, pH 7.5 1 M) containing 5% (w/v) non-fat dried milk (Sveltesse, Nestle, Spain) and 0.1% Tween-20; and incubated with the appropriate primary antibody. The antibodies employed included anti-glial fibrillary acidic protein (GFAP; BD Pharmingen, San Jose, CA, USA) was used at a concentration of 1:750, anti-cannabinoid receptor type 1 (CB1; Sigma-Aldrich, St. Louis, MO, USA) and 2 (CB2; Sigma-Aldrich, St. Louis, MO, USA) were used at a concentration of 1:500, anti-brain-derived neurotrophic factor (BDNF; Santa Cruz biotechnology, Dallas, TX, USA) and anti-synaptophysin (Sigma-Aldrich, St. Louis, MO, USA) were used at a concentration of 1:300, anti-Synaptosomal-associated protein 25 (SNAP25; AbD Serotec, Raleigh, NC, USA) was used at a concentration of 1:1000. Membranes were subsequently washed and incubated with the corresponding secondary antibody conjugated with peroxidase. Bound peroxidase activity was visualized by chemiluminescence and quantified by densitometry using ImageJ software 1.43× (NIH, New York, NY, USA). All blots were rehybridized with actin to normalize each sample for gel loading variability. All data are normalized to control values on each gel.

#### Neurotransmitter and Epigenetic Markers

The tissue samples were homogenized in 250 mg tissue/0.5 ml cold lysis buffer (1% NP-40, 20 mM, Tris–HCl pH 8, 130 mM NaCl, 10 mM NaF, 10 g/ml aprotinin, 10 g/ml leupeptin, 10 mM DTT, 1 mM Na_3_VO_4_ and 1 mM PMSF). Brain homogenates were kept on ice for 30 min, centrifuged at maximum speed for 15 min, and the supernatant was collected to determine the proteins levels using the BCA Assay (Thermo Fisher Scientific Inc., Waltham, MA, USA). Lysates were separated by SDS-PAGE gels and transferred to PVDF membranes following standard techniques.

Membranes were blocked with 5% BSA in TBS containing 0.1% Tween-20 (TBS/T), and were then incubated overnight with the following primary antibodies: anti-SR2A (1:500; Santa Cruz Biotechnology), anti-DRD1 (1:2000; Santa Cruz Biotechnology), anti-DRD2 (1:1000; Santa Cruz Biotechnology), anti-pNMDAR2B (1:1000; Abcam plc.), anti-NMDAR2B (1:1000, Abcam), anti-EAAT1 (1:1000, Abcam), anti-Lys^9^-acetyl-histone H3 (1:500; Cell Signaling Technology, Hertfordshire, UK), anti-Lys^5^-acetyl-histone H4 (1:500; Cell Signaling Technology). After washing with TBS/T, blots were incubated with appropriate HRP-conjugated secondary antibody. Proteins were visualized either with alkaline phosphatase conjugate (Sigma-Aldrich) or an enhanced chemiluminescence system (ECL Plus; Thermo Fisher Scientific Inc.). To make the loading control some membranes were stripped 30 min at room temperature, washed and incubated with anti-GAPDH mAb (1:5000; Chemicon, Hampshire, UK) or anti-tubuline (1:1000, Sigma-Aldrich). The intensity of the bands was quantified with the Alpha-Ease FC program image analysis program (Alpha Innotech Corporation).

### Statistical Analyses

In general, data were analyzed by using a two-way analysis of variance (ANOVA), considering sex (Male or Female) and adolescent intermittent exposure (Control, Co or Ethanol, EtOH) as independent factors. Shapiro-Wilk and Levene tests were used to confirm normality and homocedasticity. Ethanol intake data were analyzed by a repeated measures one-way ANOVA, considering sex (male or female) as the independent factor. Additional one-way ANOVAs were employed when needed. *Post hoc* comparisons (Bonferroni or DMS) were performed in case of significant interaction between factors. *T* test comparisons were also employed in some cases. Significance level was set at *p* < 0.05. Statistical analyses were performed by the SPSS 19.0 software package (SPSS Inc., Chicago, IL, USA).

## Results

### Alcohol Intake during Adolescence

**Figure 2:**
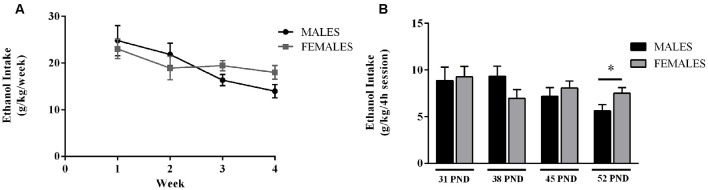
Ethanol consumption during adolescence. Data are presented as mean ± SEM. **(A)** Weekly ethanol intake (g/kg/week). **(B)** Average amount of ethanol consumed during the last 4 h session, on the 4th day of each week (g/kg/4 h session). *Wistar* rats were given access to ethanol (20% in drinking water) or tap water for 2-h sessions during 3 days, and for an additional 4-h session on the 4th day, every week during adolescence, from postnatal days (pnd) 28–52. *n* = 24 animals per experimental group, coming from 12 litters. *p* < 0.05; *effect of sex.

Alcohol intake values are shown in Figure 2. No sex differences in weekly alcohol consumption were found (Figure 2A). However, a trend for a sexual dimorphism arose by the 3rd and 4th week (*p* = 0.063; and *p* = 0.057, respectively). By the end of the alcohol exposure procedure female animals seem to consume higher amounts of alcohol than their sibling males. Actually, a significant effect of sex was observed on the last 4 h session, on pnd 52 (*F*_(1,46)_ = 4.38; *p* = 0.042; Figure 2B). By the end of the alcohol administration protocol female rats seemed to consume higher amounts of alcohol than male animals; despite this profile was the opposite at the beginning of the administration protocol: males drinking more alcohol than females during the first week. This inversion in the pattern of alcohol consumption is in agreement with previous literature showing that at adolescence, males consume more ethanol than females, whereas adult females generally exhibit higher ethanol intake than their male counterparts (Cailhol and Mormède, 2001; Vetter-O’Hagen et al., 2009).

Data from weekly alcohol (and water) consumption during the drinking sessions were also analyzed. Both male and female animals preferred water to alcohol during the exposure protocol (Amount of fluid intake (mL/kg) during the 2 h sessions in males: water: 44.88 ± 4.21 mL/kg, and alcohol: 30.43 ± 3.44 mL/kg; and in females: water: 51.72 ± 5.03 mL/kg and alcohol: 31.35 ± 3.10 mL/kg).

Ethanol intake data could also be influenced by the fact that food was not available during the DID session (2–4 h); thus, animals could be prompted to drink because of the caloric power of ethanol. However, if food was available drinking could have been enhanced through eating. Future experiments should take all these factors into account to better understand animal models of human alcohol consumption.

### Blood Ethanol Concentration (BEC)

After the last 4 h alcohol exposure session (pnd 52) BEC did not differ between male and female animals, values were 24.66 ± 1.80 mg/dL in male rats and 22.77 ± 2.01 mg/dL in female rats.

### Evaluation of Alcohol Withdrawal Signs following Alcohol Cessation

On pnd 53, 24 h after last alcohol exposure, no effects of adolescent alcohol were observed on plasma corticosterone levels (*F*_(1,24)_ = 1.02; n.s.), but a significant effect of sex was revealed (*F*_(1,24)_ = 26.65; *p* < 0.001). As expected, females showed higher plasma corticosterone levels than males (Control: males 295.42 ± 52.74 vs. females 473.66 ± 71.46; Alcohol: males 190.31 ± 8.61 vs. females 521.31 ± 77.98). Similarly, on the EPM, no changes in anxiety-like responses were observed due to the adolescent alcohol exposure (Table 1).

**Table 1:** Elevated plus maze.

	MALES		FEMALES	
	Control	Ethanol	Control	Ethanol
Open arm entries (%)	31.27 ± 5.77	22.63 ± 5.69	27.64 ± 4.92	29.99 ± 6.55
Time in open arms (%)	31.63 ± 7.29	21.76 ± 6.19	27.83 ± 5.51	31.80 ± 7.74
Closed arm entries (nr.)	7.86 ± 0.77	10.09 ± 0.72	8.46 ± 0.69	8.83 ± 0.75

*Wistar rats were given access to ethanol (20% in drinking water) or tap water for 2-h sessions during 3 days, and for an additional 4-h session on the 4th day, every week during adolescence, from pnd 28–52. After (pnd 54), animals were submitted to the elevated plus maze, 5 min session. Data are presented as mean ± SEM. *n* = 13–15 animals per experimental group, coming from eight litters*.

### Cognitive Function

**Figure 3:**
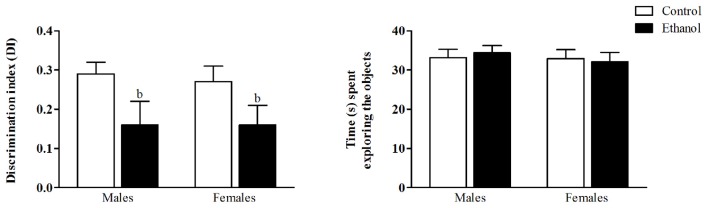
Novel Object Recognition test (NOR). *Wistar* rats were given access to ethanol (20% in drinking water) or tap water for 2-h sessions during 3 days, and for an additional 4-h session on the 4th day, every week during adolescence, from pnd 28–52. At adulthood (pnd 63), animals were submitted to the NOR test. The discrimination index (DI) was calculated as the difference between the time spent exploring the novel object (N) and the familiar one (F1 or F2) in relation to the total time spent exploring the objects ((N − F)/(N + F)). Data are presented as mean ± SEM. Analysis of variance (ANOVA), ^b^*p* < 0.05, general effect of treatment. *n* = 13–15 animals per experimental group, coming from eight litters.

As young adults, animals were evaluated for their recognition memory in the NOR test. During the training phase, no differences in total exploration times were observed (data not shown). During the test phase, the two-way ANOVA revealed a significant main effect of the alcohol condition on the DI (*F*_(1,51)_ = 6.20; *p* < 0.05). The animals exposed to the alcohol DID protocol during the adolescent period exhibited diminished discrimination values, thus indicating an impaired ability to recognize the novel object. No effects of sex was found (*F*_(1,51)_ = 0.43; n.s.), nor a significant interaction between factors (*F*_(1,51)_ = 0.01; n.s.). The total time animals devoted to the exploration of both objects did not differ between groups (Figure 3).

### GFAP Expression Levels

**Figure 4:**
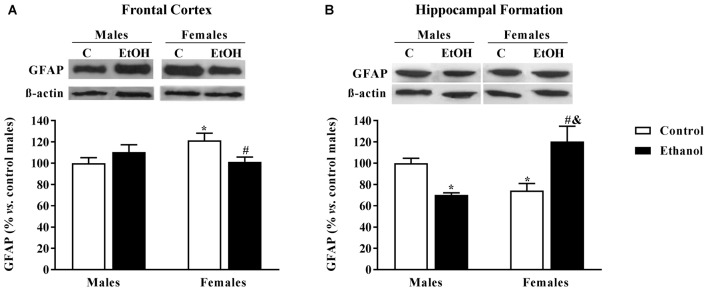
Glial Fibrillary Acid Protein (*GFAP*) expression, as an astrocyte marker. Protein expression levels within the **(A)** frontal cortex (FC) and **(B)** Hippocampal formation (HF) of adult male and female rats that were exposed to a ethanol (20% in drinking water) or tap water for 2-h sessions during 3 days and for an additional 4-h session on the 4th day, every week during adolescence, from pnd 28–52. Histograms (mean ± SEM) represent the protein levels expressed as values of optical density calculated as changes from the control male group (%); representative western blotting bands are presented above each histogram. ANOVA, *p < 0.05 vs. control male; ^#^*p* < 0.05 vs. control female; ^&^*p* < 0.05 vs. alcohol male. *n* = 6 per experimental group, coming from four litters.

In the FC, a significant interaction between sex and alcohol exposure was found (*F*_(1,20)_ = 6.59; *p* < 0.05); a basal sexual dimorphism was revealed with females showing higher levels of GFAP than males, and alcohol seemed to exclusively affect female animals decreasing its expression levels (Figure 4A). In the HF a significant interaction between factors was also reported (*F*_(1,20)_ = 20.97; *p* < 0.005). In this occasion, females showed lower GFAP levels than males, and alcohol induced an opposite effect on male and female animals: GFAP expression levels were increased among alcohol-exposed males while diminished among females (Figure 4B).

### CB1R and CB2R Expression

**Figure 5:**
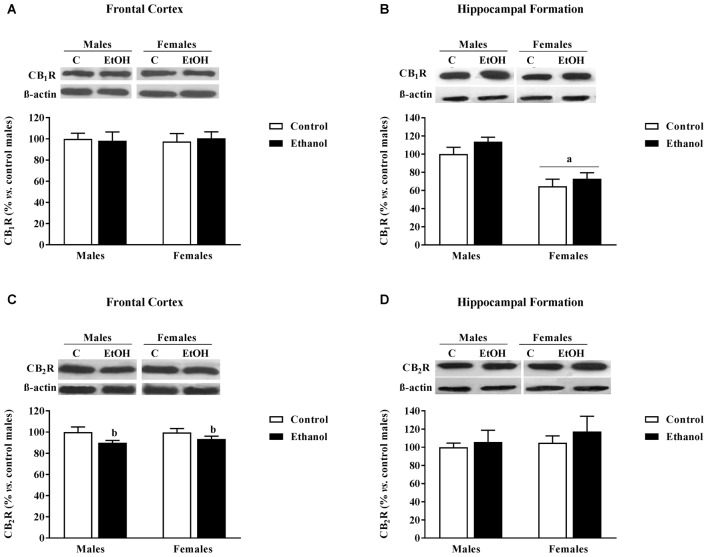
Cannabinoid Receptors (CB1R and CB2R) expression. Protein expression levels within the **(A,C)** FC and **(B,D)** HF of adult male and female rats that were exposed to a ethanol (20% in drinking water) or tap water for 2-h sessions during 3 days, and for an additional 4-h session on the 4th day, every week during adolescence, from pnd 28–52. Histograms (mean ± SEM) represent the protein levels expressed as values of optical density calculated as changes from the control male group (%); representative western blotting bands are presented above each histogram. ANOVA, ^a^*p* < 0.05 general effect of sex; ^b^*p* < 0.05 general effect of treatment. *n* = 6 per experimental group, coming from four litters.

In the FC no significant effects on CB1R expression were found (Figure 5A). However, there was a significant effect of alcohol adolescent exposure on CB2R expression (*F*_(1,20)_ = 5.46; *p* < 0.05), with CB2R levels diminished in the alcohol exposed groups compared to their corresponding counterparts (Figure 5C).

In the HF, a significant main effect of sex was found on CB1R expression (*F*_(1,20)_ = 31.47; *p* < 0.005) with females showing lower expression levels than males (Figure 5B). No significant effects were observed on CB2R expression (Figure 5D).

### Synaptic Plasticity Markers

**Figure 6:**
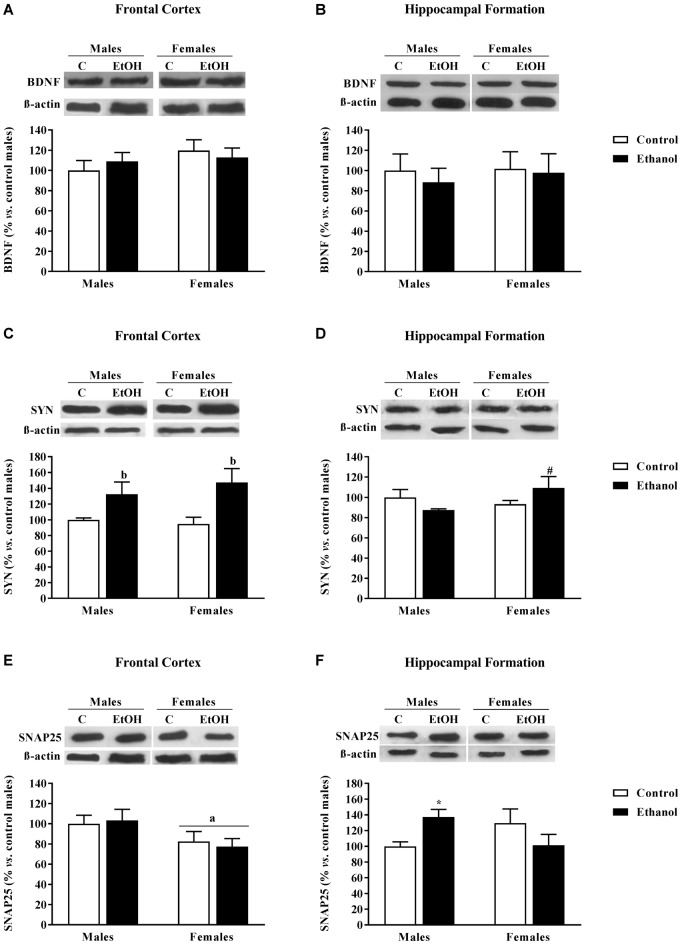
Expression of some markers of synaptic plasticity. Protein expression levels of **(A,B)** brain derived neurotrophic factor (BDNF); **(C,D)** synaptophysin (SYN); and **(E,F)** SNAP25 within the frontal cortex (left panels, **A,C,E**) and the Hippocampal Formation (right panels, **B,D,F**) of adult male and female rats that were exposed to ethanol (20% in drinking water) or tap water for 2-h sessions during 3 days, and for an additional 4-h session on the 4th day, every week during adolescence, from postnatal days (pnd) 28–52. Histograms (mean ± SEM) represent the protein levels expressed as values of optical density calculated as changes from the control male group (%). ANOVA, ^a^*p* < 0.05 general effect of sex; ^b^*p* < 0.05 general effect of treatment. *p < 0.05 vs. control male; ^#^*p* < 0.05 vs. control female. *n* = 6 per experimental group, coming from four litters.

No changes in BDNF expression levels were detected in the FC (Figure 6A) or in the HF (Figure 6B).

Synaptophysin (SYN) expression levels were affected by adolescent alcohol exposure. In the FC, the ANOVA rendered a significant effect of alcohol exposure (*F*_(1,20)_ = 11.80; *p* < 0.005); adolescent alcohol increased SYN levels in both male and female animals (Figure 6C). In the HF, a significant interaction between factors was observed (*F*_(1,20)_ = 4.08; *p* < 0.06), only among alcohol-exposed females were SYN levels augmented (Figure 6D).

Regarding SNAP25, in the FC, a significant effect of sex was found (*F*_(1,20)_ = 5.81; *p* < 0.05); females exhibited lower SNAP25 levels than male animals (Figure 6E). In the HF a significant interaction between sex and alcohol exposure was found (*F*_(1,20)_ = 5.87; *p* < 0.05), in this case, alcohol exposed males demonstrated an increase in SNAP25 expression levels (Figure 6F).

### Neurotransmitter Markers

**Figure 7:**
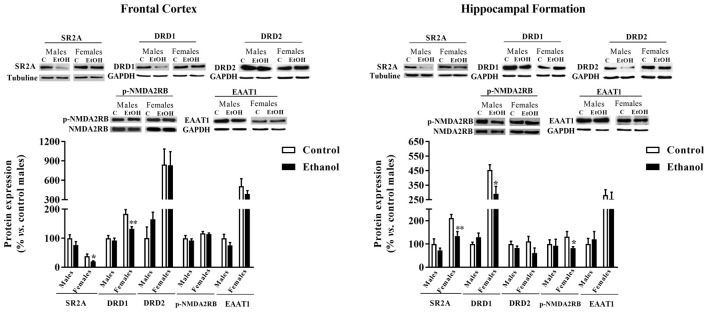
Expression of some markers of serotoninergic, dopaminergic and glutamatergic neurotransmission. Protein expression levels of serotoninergic receptors (5HT2A y 5HT2B), dopaminergic receptors (D1R and D2R), pNMDA2RB and EAAT1 in the FC (left panels) and the HF (right panels) of adult male and female rats that were exposed to a ethanol (20% in drinking water) or tap water for 2-h sessions during 3 days, and for an additional 4-h session on the 4th day, every week during adolescence, from pnd 28–52. Histograms (mean ± SEM) represent the protein levels expressed as values of optical density calculated as changes from the control male group (%). *t*-Student, *p < 0.05, **p < 0.01 compared with the corresponding control group. *n* = 5–8 per experimental group, coming from four litters.

In the FC (Figure 7), significant decreases in both the serotonergic SR2A and the dopaminergic DRD1 expression were observed among female animals (*p* = 0.040 and *p* = 0.009, respectively) as a consequence of adolescence alcohol exposure.

A similar effect of adolescent alcohol administration was observed within the HF (Figure 7). A significant decreased in the expression of SR2A (*p* = 0.007) and DRD1 (*p* = 0.028) was observed. In the HF a significant difference between adolescent alcohol exposed females was also reported for the p-NMDAR2B expression compared to control females (*p* = 0.036).

### Epigenetic Markers

**Figure 8:**
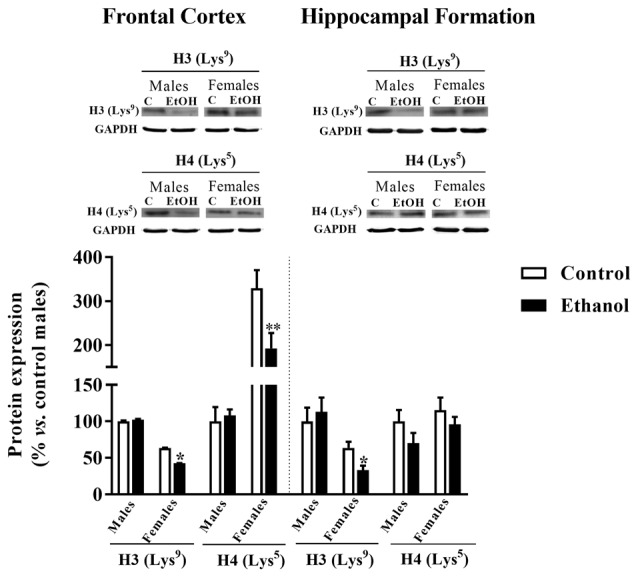
Epigenetics markers. Protein expression levels of H3 (Lys^9^) and H4 (Lys^5^) in the FC (left panels) and the HF (right panels) of adult male and female rats that were exposed to a ethanol (20% in drinking water) or tap water for 2-h sessions during 3 days, and for an additional 4-h session on the 4th day, every week during adolescence, from pnd 28–52. Histograms (mean ± SEM) represent the protein levels expressed as values of optical density calculated as changes from the control male group (%). *t* Student, *p < 0.05, **p < 0.01 compared with the corresponding control group. *n* = 5–8 per experimental group, coming from four litters.

A reduction in H3 (Lys^9^) was observed between alcohol-exposed females and their corresponding control group within the FC (*p* = 0.0004) and the HF (*p* = 0.031). Similarly, a decrease in H4 (Lys^5^) expression was only observed due to adolescent alcohol exposure among females in the FC (*p* = 0.021) but not in the HF (Figure 8).

## Discussion

In the present study, by using the DID schedule, we aimed to mimic the most common pattern of alcohol consumption at adolescence, the so-called “binge drinking”. Alcohol consumption data are highly variable as previously demonstrated in a similar paradigm (Momeni and Roman, 2014). We achieved alcohol consumption data similar to those presented in previous studies using a similar DID schedule in adult *Wistar* rats: consumption data of about 3.6 g/kg of ethanol for an access time of 2 h has been previously reported (George et al., 2012), although Holgate et al. (2017) reported a lower mean value of 1.83 g/kg/4 h session and 8.94 g/kg/week. Other studies in which access to alcohol was allowed for 24 h reported alcohol consumption of around 5.2 g/kg ethanol (Cippitelli et al., 2012) or 5.8 g/kg (Simms et al., 2008), data that can also be taken into account since previous data have suggested that “*rats intermittently exposed to the 20% alcohol solution voluntarily drank in bouts of sufficient size and frequency particularly during the first hour following the onset of the exposure*” (Cippitelli et al., 2012). Based upon BEC levels, our animals did not achieve the criterion (BECs at or above 80 mg/dL). Recently, Holgate et al. (2017) have proposed alternative binge criteria more specific for rodents; they consider that, if one standard drink contains 14 g of pure alcohol and the average body weight of an American adult is 80.7 kg, then, an average adult male rat would need to consume around 0.87 g/kg of ethanol in 2 h to meet the criteria of binge drinking. Taking this new proposal into consideration, our rats did consume alcohol at a binge-drinking rate, although BEC could not give support to this assumption. Controversial data may result from the higher metabolic rate and smaller body size of rats (compared to humans) would make it difficult for the rats to consume enough ethanol to reach the BEC of 80 mg/dL defined for humans (Holgate et al., 2017). Moreover, it has been reported that *Wistar* rats need to consume higher amounts of ethanol than other strains (e.g., Long–Evans rats) to reach the same BECs (Simms et al., 2008; Carnicella et al., 2014). In addition, BEC were evaluated 90 min after last alcohol session based upon literature (McClain et al., 2011), although this point may not reflect the maximal peak of alcohol absorption in this alcohol drinking paradigm; other studies have rather suggested a 30 min time to be more appropriate for blood sampling (Cippitelli et al., 2012). Moreover, each animal may have followed a specific pattern of alcohol consumption during the 4 h session, thus increasing the variability of BEC data. A more exhaustive analysis of alcohol consumption during the last alcohol session might be needed to be able to characterize the time course analysis of BEC in male and female adolescent animals (possibly different to that of adults), among which, pharmacodynamic and metabolic differences might be present.

No signs of alcohol withdrawal were observed in our adolescent animals following intermittent access to alcohol. In line with our findings, a previous study reported no signs of elevated anxiety at adolescence short after a single large dose of ethanol, while an acute withdrawal reaction was observed among adults (Doremus et al., 2003). Indeed, adolescent rats have been described as less vulnerable than adults to some acute effects of alcohol such as sedation, motor alteration and acute withdrawal (Little et al., 1996; Silveri and Spear, 1998; White et al., 2002; Varlinskaya and Spear, 2004). The anxiety-related symptoms observed among adults during hangover may serve as a deterrent for alcohol drinking, instead, its lack at adolescence may increase the risk for alcohol drinking behaviors at this age, thus facilitating the perpetuation of cycle of drinking that may lead to dependency and possibly alcohol-related problems at adulthood. In addition, a higher sensibility to ethanol-induced rewarding properties have been described among adolescents (Doremus-Fitzwater et al., 2010). Therefore, adolescents might be at great risk for alcohol consumption. Is it worth mentioning that among adult animals, a similar alcohol DID schedule rendered no effects on the elevated plus-maze (Cippitelli et al., 2012). Besides, controversial results have been reported following repeated binge-drinking during adolescence: increased anxiety levels have been reported, yet in the long-term (Rasmussen et al., 2001; Montesinos et al., 2016; Sanchez-Marin et al., 2017), and also decreased anxiety or increased impulsivity has also been reported (Gilpin et al., 2012). Our data may reflect moderate alcohol consumption during adolescence, a dampen effect of alcohol when administered by a DID protocol, or even resilience to the anxiogenic-like effects of alcohol at adolescence. Further studies are needed to clarify this point.

According to previous literature by using the same (Sanchez-Marin et al., 2017) or other behavioral paradigms (Coleman et al., 2014; Oliveira et al., 2015), our results further support the detrimental effects of adolescent alcohol in cognitive function (Guerri and Pascual, 2010; Alfonso-Loeches and Guerri, 2011). Alcohol-induced deficits in cognitive function have been related to changes in the volume of the HF, the orbitoFC, cerebellum and thalamus in animals (Coleman et al., 2014; Oliveira et al., 2015) and humans (De Bellis et al., 2000; Ozsoy et al., 2013), though decreases in hippocampal volume have also been related to alcohol consumption vulnerability (Nagel et al., 2005).

In the present study, the alcohol-induced deficit in cognitive function could be related to the observed increase in SYN expression within the FC. Despite in the short-term alcohol may decrease SYN expression due to its neurotoxic effects, possibly related to neuronal excitotoxic damage resulting from repeated alcohol withdrawal episodes (Alfonso-Loeches and Guerri, 2011), a compensatory up-regulation may arise if alcohol is continued and/or administered at adolescence. Changes in presynaptic markers have also been reported in the HF: a similar increase in SYN was observed among females, although SNAP25 seems to play this role in males. We found no changes in BDNF expression, although in the literature controversial results have been reported (decreases (Briones and Woods, 2013) and increases (Tapia-Arancibia et al., 2001) in the hippocampus). Discrepancies may rely on the alcohol paradigm used, age of the brain examined, and timing of measurement during the withdrawal period. In general, repeated alcohol exposure during adolescence seems to interfere with presynaptic proteins inducing a long-lasting compensatory up-regulation that may critically affect synaptic function and the establishment of adult neural circuitries.

The endocannabioid system has also been given a role in alcohol effects (Basavarajappa and Hungund, 2002). We observed no changes in CB1R expression, but a significant decrease in CB2R expression within the FC was observed in both male and female animals. A recent study has reported a decrease in the mRNA expression of *Cnr2* in the hippocampus following adolescent alcohol administration (Sanchez-Marin et al., 2017) that has been demonstrated to modulate cognitive functions in mice (Li and Kim, 2016). On the other hand, existing literature also suggests that a reduction in CB2R function may promote alcohol preference and consumption (Onaivi et al., 2008; Ortega-Álvaro et al., 2015). Therefore, the alcohol-induced decrease in CB2R may underlay the alcohol-induced cognitive impairment, but may also be related to the reported increase for adult alcohol consumption following adolescent exposure (Guerri and Pascual, 2010).

Adolescent exposure to alcohol also impacts glial cells, and important changes in GFAP expression have been previously reported. An increase in GFAP levels have been reported short after alcohol exposure during adolescence, although in the long-term, after abstinence, levels tended to return to normality (Evrard et al., 2006; Kane et al., 2014). The effects of adolescent alcohol on glial cells remains controversial and further studies are needed to better understand alcohol sex-dependent effects, and the possible relevance of the sex and brain-region dependent maturational program of astrocytes (Koss et al., 2012).

Remarkably, intermittent access to ethanol during adolescence seems to particularly affect female animals consistently reducing molecular markers of serotoninergic, dopaminergic and glutamatergic signaling. Such an effects may reflect an increase in neuronal apoptosis, as recently described for the hippocampus (Oliveira et al., 2015) or it may reflect specific effects of the different neurotransmitter systems. Adolescent alcohol indices a loss in 5-HT immunoreactive neurons within the dorsal raphe nucleus (DRN; Evrard et al., 2006; Vetreno et al., 2017) together with persistent alterations in terminal field projections, i.e., hypothalamus, amygdala and hippocampus (Vetreno et al., 2017). Adolescent alcohol also impacts the dopaminergic system by decreasing the expression of its receptors, at least, in the medial pre-FC (Pascual et al., 2009; Trantham-Davidson and Chandler, 2015; Crews et al., 2016).

Epigenetic changes have been associated with behavior during development (Roth, 2013) and epigenetics mechanisms may also underlying the sex-specific consequences of adolescent alcohol. In the present study, exclusively among females, a decrease in H3 acetylation in the two brain regions analyzed has been evidenced, but a decrease in H4 acetylation only within the FC. However, previous studies showed that alcohol administration during adolescence increases H3 and H4 acetylation in the preFC of mice (Pascual et al., 2012; Montesinos et al., 2016). Actually, in those studies acetylation changes were related with changes in the expression of specific genes such as bdnf but also cFos, Cdk5 and FosB. Moreover, adolescent alcohol was reported to also up-regulate histone acetyl transferase (HAT) activity in the preFC. In these studies, these epigenetic changes were mainly related to alcohol-induced anxiety and to the rewarding effects of alcohol. Therefore, discrepancies with our present results may rely on differences on drug doses and routes, on the animal specie employed, but also to the fact that no changes in anxiety were observed in our hands in the present study, and alcohol-related rewarding properties were not analyzed in this study. Further research is still needed to specifically correlate alcohol-induced behavioral and epigenetic changes.

Further research is urgently needed to better understand the underlying molecular mechanisms for adolescent alcohol consequence, mainly for the possible resilience to alcohol negative effects, including withdrawal, for the long-lasting cognitive impairment, as well as for the increased vulnerability of females to alcohol (Barron et al., 2005; Spear, 2015). The consequences of alcohol seem to critically depend on the ethanol dose, administration route and schedule, peak BEC, treatment paradigm (including length of treatment and the presence or absence of a withdrawal period following ethanol administration), sex and the age of the animal (reviewed in Drew and Kane, 2013). Thus, the development of better and more consistent translational methods for the evaluation of adolescent alcohol detrimental effects is of great medical and societal concern.

In spite of the fact that prevention and alcohol control policies are yet effective tools in the reduction of excessive alcohol consumption, a better knowledge of the mechanisms involved in alcohol effects may provide new tools for the identification of vulnerability populations, and may open new horizons in the pharmacology of alcohol abuse disorders.

## Author Contributions

All authors had full access to all data in the study and take responsibility for the integrity of the data and the accuracy of the data analyses. Design of the work: FRF, ML-G and EMM. Acquisition of data: SP, M-DH, AG, EB, EG, JAL-M and EMM. Analysis of data: SP, M-DH, EB, MM, AG, EG and EMM. Interpretation of data: CG, FRF, ML-G and EMM. Drafting of the manuscript: ML-G and EMM. Critical revision of the manuscript for important intellectual content, obtained funding and study supervision: ML-G, EMM, JAL-M, CG and FRF. All authors revised the manuscript critically for intellectual content; and gave their final approval of the version to be published.

## Conflict of Interest Statement

The authors declare that the research was conducted in the absence of any commercial or financial relationships that could be construed as a potential conflict of interest.
